# Development of an instrument evaluating the impact of surgeon-patient relationship in patients on sick leave

**DOI:** 10.1080/20016689.2017.1345586

**Published:** 2017-08-11

**Authors:** Thierry Dubert, Cedric Girault, Alexandre Kilink, Marc Rozenblat, Yves Lebellec, Anne-Lise Vataire, Myriam Vilasco, Gregory Katz

**Affiliations:** ^a^ Ramsay Générale de Santé, Clinique Jouvenet, Paris, France; ^b^ Ramsay Générale de Santé, Hôpital Privé Paul d’Egine, Champigny, France; ^c^ Groupement de Coopération Sanitaire du Réseau Prévention Main Ile de France, Paris, France; ^d^ Clinique de l’Yvette, Longjumeau, France; ^e^ Creativ-Ceutical, Paris, France; ^f^ ESSEC Business School, Paris-Singapore, France; ^g^ Paris-Descartes University, School of Medicine, Chair of Innovation Management & Healthcare Performance, Paris, France

**Keywords:** Surgery, return to work, musculoskeletal disorders, hand trauma, instrument validation, patient-surgeon relationship, Value-Based Health Care, PROM

## Abstract

**Background**: To date, no specific instruments exist to measure the quality of the patient-surgeon relationship despite its potential to influence clinical and economic outcomes in patients undergoing surgery for musculoskeletal disorders (MSDs).

**Objective**: The objective was to develop and validate an instrument to assess the quality of the patient-surgeon relationship, taking into account the return to work after functional restoration surgery.

**Methods**: The instrument development was based on literature review, cognitive interviews and expert examinations. The instrument’s psychometric properties were explored in a sample of 50 French patients on sick leave with musculoskeletal disorders or hand injuries. Face validity, internal consistency and test-retest reliability were evaluated. The dimensionality of the instrument was studied using an exploratory principal component analysis.

**Results**: The 11-item instrument showed good psychometric properties. The cognitive interviews allowed enhancing the validity of the instrument content by capturing patients’ point of view. The exploratory principal component analysis demonstrated the uni-dimensionality of the instrument with the first factor accounting for 83% of the total explained variance.

**Conclusion**:This study has developed the first instrument capable of the specific assessment of the impact of the surgeon-patient relationship on recovery, in patients with hand traumas and MSDs.

## Introduction

Surgical outcomes depend primarily on the indication and the technique; but it is also well established that the quality of the relationship between the healthcare provider and the patient significantly impacts the recovery process [1–4], adherence to therapies and treatment outcomes [2,5,6], as well as patient satisfaction [5,7]. Several instruments have been developed to assess care giver-patient relationship [8]. These studies showed that the relationship quality, as perceived by the patient, is primarily determined by the healthcare provider’s good will as well as his ability to communicate [9–11]. However, none of these instruments were neither broadly applicable, nor suited for a surgical context [12,13].

As a hand and upper extremity network including surgeons, physiotherapists, occupational specialists, psychologists and social workers, we are particularly exposed to cases of severe organic, functional and relational disabilities resulting from musculoskeletal disorders (MSD). The aim of the present study was, therefore, to develop an instrument to assess patient-surgeon relationship and validate it in a sample of French patients with upper extremity MSD requiring long term sick leave, allowing formal analysis of such relationship and creating an opportunity to improve the standard of care for surgical treatment.

## Methods

A questionnaire to evaluate patient’ s perception of the surgeon-patient relationship in patients requiring long term sick leave was developed following a four step process: literature review, expert review, cognitive interviews and psychometric validation of the final instrument.

### Literature review

A search in Medline and EMBASE databases was carried out to identify literature containing existing questionnaires for assessing patient-caregiver relationship. A search algorithm was developed using the following terms: « Doctor », « Physician », « Surgeon », « Professional », « Patient », « Relation », « Test », « Survey », « Measure », « Scale », « Questionnaire », « Psychometry » and « Psychometrics ». Screening was independently conducted by two reviewers to include all articles presenting a survey or questionnaire evaluating the relationship between patients and health professionals. The search was limited to articles written in English and French. To be included, articles were required to present self-completed patient questionnaires and contain items for assessing patient-physician relationship, to present the psychometric properties of the questionnaires, and to include the final version of the questionnaire. Articles were excluded if they were restricted to children and adolescents, or if the patient-physician relationship was part of a health organization and notably different from those of surgical services (for example, psychiatric care for which the patient and the doctor are required to interact more regularly and personally).

### Instrument development

The most relevant questionnaire items identified during the literature review were selected, translated and/or adapted and assembled to form a version A of the new instrument. Version A was then reviewed by a group of experts, including the research team and two psychologists from Hand Injury Prevention Network Ile-de-France (Hand Injury Prevention Network Ile-de-France, RPMIDF [14]) who regularly hold consultations with patients with upper extremity MSD. The group’s objective was to revise the content of the questionnaire by selecting the most appropriate items for inclusion and by reviewing the wording based on possible responses. It was decided to structure each item as a statement made from the patient’s perspective with four possible responses: « Strongly disagree », « Tend to disagree », « Agree » and « Strongly agree ». A Likert-type scale with four response options was selected to avoid neutral answers. This approach was taken to limit the possibility of social desirability bias, as well as the tendency of patients to avoid negative answers for fear of the possible consequences of the expression of a negative feeling [15]. The review of the expert group resulted in a version B that was subsequently tested on patients.

Two iterative cycles of cognitive interviews were conducted face-to-face in a sample of ten patients with upper extremity MSD [16]. All interviews were conducted by a psychologist. Each patient completed the instrument by expressing his choices aloud, thereby enabling an assessment of his level of understanding of each item. At the end of the interview, each patient was asked to express his overall impression and suggest additional questions. The first round of interviews (N = 5) was designed to assess the overall understanding and relevance of the instrument in practice. The second round of interviews (N = 5) was designed to evaluate whether the questionnaire was perfectly understandable by patients after modification. Analyses of the cognitive interviews resulted in a version C of the questionnaire that was used in a larger group of patients and statistically validated.

### Instrument validation

The instrument was administered to the first 50 patients enrolled in an ongoing observational study being conducted in eight hospitals in France specialized in the management of diseases of the hand (Hand Injury Prevention Network Ile-de-France, RPMIDF [14]). The study protocol and the questionnaire (including our instrument for measuring the quality of the relationship) were previously submitted and approved by data protection regulatory bodies (on 12 March 2014 by the *Comité consultatif sur le traitement de l’information en matière de recherche* and on 14 October 2014 by the *Commission nationale de l’informatique et des libertés)*. Patients aged 18–55 years with trauma or MSD of the upper limb and regularly followed-up by a referred surgeon at the Prevention Network Main Île-de-France (RPMIdF) were included in the study. Other study inclusion criteria were: 1/tenure holder of public sector or employees with a private open-ended contract who have completed their probationary period, 2/patients who have been on sick-leave because of the trauma or MSD of the upper limb, 3/French-speaking patients. Patients with severe comorbidities requiring a sick leave, or whose sick leave was not related to upper extremity MSD were excluded from the study.

To examine the instrument properties, each patient was asked to complete the questionnaire during two separate visits, at two-week intervals. The patient’s response to each item was assigned a numerical value or a score ranging from 1 for the response « Strongly disagree » up to 4 for the response « Strongly agree ». The overall score of the instrument was obtained by adding the scores of all the items, and then normalizing the total on a scale from 0 to 100 (100 indicates the best possible quality of patient-surgeon relationship and 0 the worst).

The evaluation of the face validity was based on the analysis of missing data, floor and ceiling effects. The percentage of missing data was considered acceptable below 15% [17]. Floor and ceiling effects were considered acceptable below 50%, which corresponds to a distribution twice greater than a symmetrical distribution among the various items (i.e. 100% of responses divided by 4 possible responses multiplied by 2).

The instrument’s ability to reproduce the same results in two consecutive administrations conducted under identical conditions was also evaluated. The test-retest reliability was measured using the Pearson correlation coefficient calculated with the results of the first and second visit. The time interval between these two visits (16 ± 6 days) was sufficient to ensure that patients are unlikely to precisely remember their previous answers. No intervention was set up between the two visits that had the potential to change the patient-surgeon relationship. The interpretation of this coefficient (r_p_) was made according to the criteria defined by Donner and Eliasziw [18]. The reliability of the instrument was considered as satisfactory when a coefficient (r_p_) was greater than 0.90.

Internal consistency was estimated using Cronbach’s alpha coefficient, which reflects the homogeneity of the instrument and the complementarity of its different items. It is commonly used as a (lowerbound) estimate of the reliability of a psychometric test. An alpha coefficient greater than 0.7 is generally regarded as acceptable; while beyond 0.9 there is a possibility of redundancy between items [19]. Cronbach’s alpha is a function of the number of items in a test, the average covariance between item-pairs, and the variance of the total score. An exploratory principal component analysis was also performed to examine the dimensionality of the instrument. The choice of the number of selected factors was based on several criteria: the Kaiser-Guttman criterion that proposes retaining only the factors with ‘eigenvalue’ > 1 [20,21]; the analysis of the scree plot that proposes retaining the number of factors for which the line stops decreasing abruptly and levels out, when ‘eigenvalues’ are plotted as a function of the number of factors [22]; factor loadings of 0.50 or better [23]; and the analysis of percentages of explained variance. Scree plots typically display the eigenvalues associated with a component or factor in descending order versus the number of the component or factor, and can be used in principal components analysis and factor analysis to visually assess which components or factors explain most of the variability in the data.

## Results

The four step process of literature review, expert review, cognitive interviews and psychometric validation led to the development of an instrument consisting of a 11-item questionnaire specifically assessing patient’s perception of surgeon-patient relationship, in patients with long-term sick leave (Table 1).

**Table 1.** Instrument for the evaluation of patient-surgeon relationship (11 items) (Supplementary Table 1).

	RESPONSE			
	STRONGLY DISAGREE	TEND TO DISAGREE	AGREE	STRONGLY AGREE
1: My surgeon easily provides me with the sick leave certificates I need.	□	□	□	□
2: My surgeon avoids using medical vocabulary so I can understand.	□	□	□	□
3: I find that information is communicated properly and consistently between different professionals who take care of my condition including my surgeon (general practitioner, physiotherapist, rheumatologist, psychologist, …).	□	□	□	□
4: I am satisfied with the availability of my surgeon (in person or by phone) when I need it.	□	□	□	□
5: My surgeon tells me when I can go back to my work; or on the contrary, he tells me that I cannot go back to work.	□	□	□	□
6: My surgeon regularly informs my doctor about my care management and about the progress of my health problem.	□	□	□	□
7: My surgeon is patient when I do not understand what he says.	□	□	□	□
8: My surgeon discusses with me the conditions of my return to work.	□	□	□	□
9: My surgeon understands the impact of my pain and my disability on my mood.	□	□	□	□
10: I’m satisfied with the time allotted to me by my surgeon during consultation.	□	□	□	□
11: My surgeon encourages me to talk about my concerns and listens to me carefully.	□	□	□	□

For publication purpose, this is an English translation of the instrument originally developed in French.

**Figure 1.**
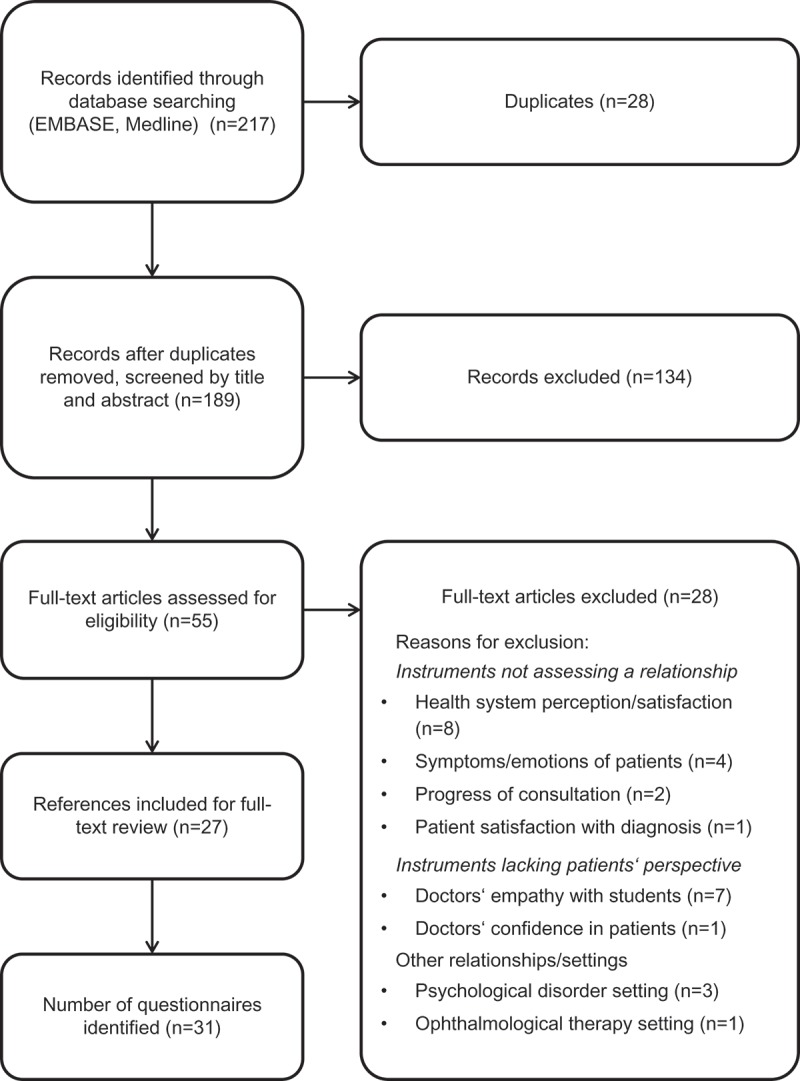
Literature review screening flow chart.

The process was initiated by a literature review, with database searches yielding a total of 217 results. Following removal of duplicates (n = 28), a total of 189 references published between 2010 and 2013 were eligible for a first round of screening (Figure 1). A further 134 references were excluded based on the screening of titles and abstracts, leaving 55 references for full-text review. In the final selection phase, 27 publications were included with details of 31 psychometric questionnaires.

Of the 31 identified questionnaires, six were validated in psychological and psychiatric fields and 25 in other medical fields such as oncology, palliative care, general medicine and alternative medicine. For the 31 instruments reviewed, Cronbach’s alpha scores were between 0.85 and 0.95 indicating that the questionnaires were generally valid for their specific uses. A total of eight questionnaires were identified that specifically targeted patient-physician relationship among which four key aspects were captured: (i) the patient’s confidence in his physician [24–26]; (ii) moral support given to the patient by the physician [27]; (iii) the projection into the future and support for the patient [28–30]; (iv) administrative cooperation [31]. No questionnaires were identified that sought to evaluate the patient-surgeon relationship.

Based on a review of existing questionnaires and identification of the most relevant items regarding communication skills and good will perception, the version A questionnaire was examined by a group of experts to develop a working version for refinement and validation with patients. Key criteria for inclusion in the version B instrument were items evaluating help offered by healthcare providers in establishing targets for return to work, especially those related to the length of the sick leave and the conditions of returning to work. Items were included if related to patient daily living issues, moral support, future projection, and surgeon cooperation in administrative issues. The adaptation of specific items was, at times, challenging especially for items from instruments used in oncology or in the context of psychological disorders. Typically, the patient-physician relationship in these therapy areas is intrinsically different from the patient-surgeon relationship, with the latter typically being over a longer term with more frequent contact. Following expert review, version B of the instrument for testing with patients included 23 items.

The first set of cognitive interviews was used to determine which items were missing, redundant, satisfactory or relevant from the patient’s perspective. Nine items were considered satisfactory, five were deleted or merged and four were reformulated (Table 2). Based on patients’ suggestions, two items were added to the instrument, one exploring surgeon’s empathy perceived by patients with the use of appropriate vocabulary, the other exploring the level of patience that the surgeon exhibits in case of misunderstanding. The second set of cognitive interviews confirmed the relevance of the items with the exception of three items that had posed problems during the first series and these were removed (Table 2). At the end of the cognitive interviews, 17 items were included in the version C instrument.

**Table 2.** List of instrument modifications based on the cognitive interviews.

First set of interviews	
**Removal**	**Reasons for removal**
« My surgeon easily writes letters for my occupational physician »	Patients do not feel concerned
« My surgeon is able to see when I’m not emotionally okay »	Redundant with « My surgeon understands the impact of my pain and my disability on my mood »
« Overall, I totally trust my surgeon. »	Redundant with « I fully trust the decisions taken by my surgeon on the management of my disease. If my doctor tells me something, he is necessarily right. »
**Modification**	**Items replaced by**
« My surgeon informs me of administrative procedures that I have to perform »	« My surgeon informs me about administrative procedures related to my health problem »
« My surgeon informed me of an estimate of the total duration of my sick leave »	« My surgeon tells me about the date I would be able to go back to my work. Or on the contrary he tells me that I could not go back to work. »
« My surgeon is optimistic /My surgeon is confident /My surgeon believes in my progress »	« Regarding my recovery, my surgeon remains positive and optimistic. »
« My surgeon helps me with my administrative procedures (health insurances.). »	Patients consider that it is not the role of the surgeon, was revised « My surgeon speaks with me about the administrative procedures that I have to perform (health insurances).
**Merger and modification**	**Items replaced by**
« My surgeon encourages me to talk about my concerns. »	« My surgeon encourages me to talk about my concerns and listens to me carefully »
« My surgeon listens to me carefully when I want to say something »	
« My surgeon describes to me the different steps of my follow-up/care management »	« Regularly we take stock together with my surgeon on the progress already made and that still to be achieved. »
« My surgeon sets my goals »	
Addition	
« My surgeon avoids using medical vocabulary so I can understand. »	
« My surgeon is patient when I do not understand what he says »	
**Second set of interviews**	
**Removal**	**Reasons for removal**
« My surgeon is reassuring on my recovery »	Redundant items and not well understood by patients
« Regarding my recovery, my surgeon remains positive and optimistic. »	
« My surgeon discusses with me the administrative procedures that I have to perform (social security, mutual, provident, etc.). or “My surgeon and /or assistant (s) discuss with me the administrative procedures that I have to perform (social security, mutual, provident, etc.) »:	As in the first set, the ten patients feel that it is not the role of the surgeon
**Minor modification**	**Items replaced by**
« Regularly we take stock together with my surgeon on the progress already made and those still to be made. »	« We take stock regularly with my surgeon on the progress already made and that still needs to be achieved. »
« My surgeon tells me when I can go back to my work; or on the contrary, he tells me that I cannot go back to work. »	« My surgeon tells me about the date I would be able to go back to work. Or on the contrary he tells me that I cannot go back to work. »

**Figure 2.**
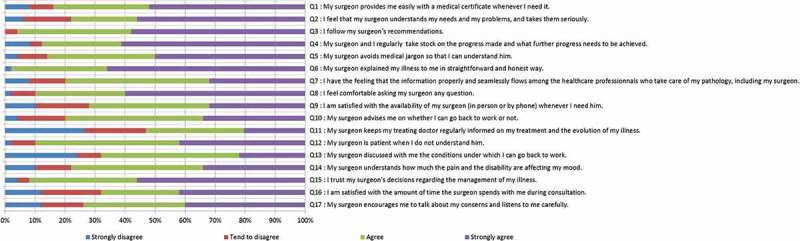
Distribution of item responses of the 17-item instrument (version C) (17 items).

The version C instrument was tested in a cohort of 50 patients in an observational study. The patients’ mean (standard deviation, SD) age was 38.3 (10.8) years and 60% of these were male. The sample of workers consisted of 62% with mainly intellectual activities, 32% with mainly manual activities and 6% managers (workers with management responsibilities).The main reason for surgery was trauma (82%) while the minority was due to MSD (18%). The mean (SD) overall score for the instrument was 67.22 (22.53) out of 100, with almost all items answered in full (there were two items for which the missing data rates were 2%). For almost all individual items, the distribution of responses was asymmetric, indicating generally high satisfaction levels (Figure 2). However, more heterogeneous distributions were observed in some items such as *«my surgeon regularly informs my doctor about my care management and about the progress of my health problem»* and items related to work *«their surgeon had discussed with them the conditions of their return to work», «my surgeon easily gives me the sick notes that I need»* and *«my surgeon tells me when I can go back to my work; or on the contrary, he tells me that I cannot go back to work»*. Seven items out of 17 had a notably skewed distribution due to the ceiling effect associated with over 50% of patients responding *«strongly agree»* (Figure 2). These seven items were removed from the instrument with the exception of the item *«my surgeon easily gives me the sick notes that I need»*, which was considered worthy of retention because no other item provides similar information.

The exploratory principal component analysis indicated that only one factor was associated with a high level of variance. The eigenvalue analysis showed that there was only one factor (the first) associated with an eigenvalue greater than 1 (5.38). The analysis of the scree plot confirmed this observation. All items had factor loadings above 0.50 on the first factor with the exception of the item *«My surgeon easily gives me the medical certificates I need»*. The first factor accounted for 83% of the total explained variance. The internal consistency of the instrument was satisfactory with a Cronbach’s alpha of 0.90. For version C, prior to the removal of 7 items from the questionnaire, Cronbach’s alpha was 0.93. The mean ± SD of intervals between two patient’s instrument completions was 16 ± 6 days. The reliability of the final instrument was satisfactory, with an intra-class Pearson’s correlation coefficient of 0.86.

## Discussion

In this study, we developed the first instrument specifically assessing patients’ perception of surgeon-patient relationship, in patients on a long-term sick leave. Given the challenges involved in the return-to-work process after a functional restoration surgery, the instrument has been designed to explicitly take into account the preparation for return to work. The validation of the instrument’s psychometric properties was an essential step towards obtaining an appropriate tool for measuring the impact of the patient-surgeon relationship on return to work. This instrument was developed following scientifically rigorous methodology for the development of patient reported outcome (PRO) instruments, including a content evaluation by the research team and an assessment of the understanding of patients surveyed by cognitive interviews [32]. As for the evaluation of the instrument’s psychometric properties, it was conducted on a sample of 50 patients according to the classical test theory [33,34].

The population of patients with upper extremity MSD was considered suitable for testing our instrument because of the availability of a large number of working age patients. MSD cover all injuries, joint pain or damage to other tissues that support the upper and lower limbs, neck and back. The upper limbs represent the main location of these pathologies, particularly those of the shoulders, elbows, carpal tunnels, wrists and fingers [35]. Carpal tunnel syndrome represents just fewer than half of all MSDs of the upper limbs [36]. Not only do upper extremity MSD impose a significant burden on public health [37–39], they are also a source of serious functional impairments, reducing the quality of life of patients and limiting their activities, in particular professional activities [40–44], and are associated with a substantial amount of sick leave and considerable loss of productivity [37,45–47]. Successful return to work following surgical intervention for upper extremity MSD is therefore an important goal for patients, and is associated with significant economic benefits for society as a whole. Interactions between patients and surgeons may therefore play an important role, both clinically and economically, in terms of ensuring successful outcomes and a rapid return to work [48–50]. The statistical analyses supported the good psychometric properties of the instrument and its validity. Cognitive interviews have added two important items for patients *«My surgeon avoids using the medical vocabulary so that I can understand»* and *«My surgeon is patient when I do not understand what he says to me»*, which enhanced the validity of the instrument content. The importance of involving patients in the design of self-evaluation questionnaires has already been emphasized in several fields [51] including hand surgery [52]. The distributions of responses for these two items have confirmed their importance to patients since the responses were not homogeneous. Similarly, distributions of responses of items specific of return to work showed the importance of these items *«My surgeon tells me when I can go back to my work; or on the contrary, he tells me that I cannot go back to work»*, and *«My surgeon has discussed with me the conditions of my return to work»*. The low rate of missing data confirmed the face validity of the instrument confirming the proper understanding of the items. Analysis of floor and ceiling effects led to the removal of six items that did not provide information since the patient responses were almost homogeneous. The other quantitative analyses support the construct validity and reliability of the instrument, with the Cronbach alpha and the intra-class correlation coefficient satisfying the usual criteria. Factor analysis showed the uni-dimensionality of the instrument with a great percentage of explained variance.

A number of limitations of this study are worth noting to properly establish context. The generalization of the psychometric results of the instrument can be limited because, although this assessment has been conducted on a reasonable number of patients, they were recruited in specialized hospitals and only in Ile de France (French region). Although there is no evidence to suggest differences in the relationships between patients and surgeons in non-specialized centres and in centres in other regions, this aspect may require further investigation. Additionally, no exploratory interviews were carried out to ensure that the underlying conceptual framework included all relevant concepts. However, patients were asked during the cognitive interviews if any concept that was relevant in their relationship with their physician was missing. Furthermore, the external validity could not be evaluated because we lacked sufficient information to make a priori hypotheses about the patients’ characteristics which would be or not be correlated with the measured score.

This instrument may be useful for health professionals committed to improving surgical treatment outcomes, both in terms of quality as well as the cost-effectiveness of surgical procedures. In the same way surgeons evaluate outcomes of surgical interventions using clinical scores; the quality of the relationship with patients could be measured with the help of this self-administrated questionnaire. The instrument should also be of interest for primary and secondary payers, and for healthcare managers in public and private health organisations for the same goals of improving quality and cost-effectiveness of healthcare.

## Conclusion

This study presents the first instrument that assesses the patient’s perception on patient-surgeon relationship. Although the internal validity of the instrument has been demonstrated, further studies should be conducted to assess the sensitivity of the instrument to change and external validity. Moreover, it would be interesting to explore the properties of this instrument in a larger population, including other pathologies and other surgical specialties (cancer surgery, abdominal surgery, vascular, neurosurgery). The development and validation of this instrument constituted a preliminary step that was needed to get an appropriate tool for measuring in a subsequent step the impact of patient-surgeon relationship on return to work after surgery.

## Supplementary Material

Questionnaire_surgeon-patient-relationship.docx
