# Supplementary material for: Development of an instrument evaluating the impact of surgeon-patient relationship in patients on sick leave
Source: J Mark Access Health Policy. 2017 Aug 11;5(1):1345586. doi: 10.1080/20016689.2017.1345586 (PMC5629953; doi:10.1080/20016689.2017.1345586)
Supplement: Questionnaire_surgeon-patient-relationship.docx [file zjma_a_1345586_sm3525.docx]

**Questionnaire d’évaluation de la relation**

**chirurgien-patient**

**Merci de lire les consignes attentivement**

Le but de ce questionnaire est d’évaluer la qualité de la relation entre le patient et son chirurgien depuis le début la prise en charge par celui-ci. Pour chacune des affirmations, merci de choisir la réponse qui vous correspond le mieux.

Quand vous répondez à une question, pensez à votre propre relation avec votre chirurgien et gardez à l’esprit qu’il n’y a pas de bonne ou de mauvaise réponse.

|  | Réponse | | | |  |
| --- | --- | --- | --- | --- | --- |
|  | Pas du tout d’accord | Plutôt pas d’accord | D’accord | Complètement d’accord | |
| 1 : Mon chirurgien me procure facilement les certificats médicaux dont j’ai besoin. | ❑ | ❑ | ❑ | ❑ | |
| 2 : Mon chirurgien évite d’utiliser du vocabulaire médical afin que je puisse comprendre. | ❑ | ❑ | ❑ | ❑ | |
| 3 : Je trouve que l'information circule correctement et de manière cohérente entre les différents professionnels qui s'occupent de ma pathologie y compris mon chirurgien (médecin généraliste, kinésithérapeute, rhumatologue, psychologue,...). | ❑ | ❑ | ❑ | ❑ | |
| 4 : Je suis satisfait(e) de la disponibilité de mon chirurgien (en personne ou par téléphone) quand j’en ai besoin. | ❑ | ❑ | ❑ | ❑ | |
| 5 : Mon chirurgien me dit quand je pourrai reprendre mon travail ; ou au contraire, il me dit que je ne pourrai pas reprendre mon travail. | ❑ | ❑ | ❑ | ❑ | |
| 6 : Mon chirurgien informe régulièrement mon médecin traitant sur ma prise en charge et sur l’évolution de mon problème de santé. | ❑ | ❑ | ❑ | ❑ | |
| 7 : Mon chirurgien est patient lorsque je ne comprends pas ce qu’il me dit. | ❑ | ❑ | ❑ | ❑ | |
| 8 : Mon chirurgien a discuté avec moi des conditions de mon retour à un travail. | ❑ | ❑ | ❑ | ❑ | |
| 9 : Mon chirurgien comprend l’impact de mes douleurs et de mon handicap sur mon moral. | ❑ | ❑ | ❑ | ❑ | |
| 10 : Je suis satisfait(e) du temps qui m'est accordé par mon chirurgien lors de la consultation. | ❑ | ❑ | ❑ | ❑ | |
| 11 : Mon chirurgien m’encourage à parler de mes inquiétudes et m’écoute attentivement. | ❑ | ❑ | ❑ | ❑ | |
|  |  |  |  |  | |
